# The Epidemiology of Bloodstream Infections and Antimicrobial Susceptibility Patterns: A Nine-Year Retrospective Study at St. Dominic Hospital, Akwatia, Ghana

**DOI:** 10.1155/2019/6750864

**Published:** 2019-09-19

**Authors:** John Gameli Deku, Mavis Puopelle Dakorah, Sylvester Yao Lokpo, Verner N. Orish, Francis Abeku Ussher, Godsway Edem Kpene, Vida Angmorkie Eshun, Eunice Agyei, Waldermer Attivor, James Osei-Yeboah

**Affiliations:** ^1^Department of Medical Laboratory Sciences, School of Allied Health Sciences, University of Health and Allied Sciences, Ho, Ghana; ^2^Laboratory Department, Cape-Coast Teaching Hospital, Cape-Coast, Central Region, Ghana; ^3^Department of Microbiology and Immunology, School of Medicine, University of Health and Allied Sciences, Ho, Ghana; ^4^Faculty of Health and Allied Sciences, Koforidua Technical University, Koforidua, Eastern Region, Ghana; ^5^Laboratory Department, St. Dominic Hospital, Akwatia, Eastern Region, Ghana

## Abstract

**Background:**

Bloodstream infections are among the top causes of morbidity and mortality in people of all ages, especially in immunocompromised patients in sub-Saharan Africa. This study aimed at describing the epidemiology of bloodstream infections and antimicrobial susceptibility pattern over a nine-year period at St. Dominic Hospital, Akwatia, in the Eastern Region of Ghana.

**Method:**

This study retrospectively analysed data from 4,489 patients who were referred to the Laboratory Department for blood culture and sensitivity testing from January 2009 to December 2017. Sociodemographic data included age, gender, and patients' department. Blood culture results were retrieved from archival records in the laboratory. The authorities of St. Dominic Hospital granted approval for the study.

**Results:**

The incidence of bloodstream infection over the 9 years was 51.4 positive cultures per 100,000 hospital attendance. *Staphylococcus aureus* was the leading causative agent of bacteraemia for the first two scalar years (2009–2011 (38.9%) and 2012–2014 (42.2%)) while coagulase-negative staphylococcus (CoNS) (50.5%) was predominant for the last scalar year (2015–2017), followed by *Staphylococcus aureus* (169/587 (28.8%)). The highest incidence of bloodstream infections was recorded in the wet seasons (months of May (8.9 per 10,000 persons) and October (10.1 per 10,000 persons)). The bacterial isolates demonstrated high resistance to tetracyclines (390/531 (73.4%)), penicillins (1282/1669 (76.8%)), and sulphonamides (450/499 (90.2%)).

**Conclusion:**

Bloodstream infection and antimicrobial resistance are high in patients seeking healthcare in Akwatia. This therefore calls for concerted efforts aimed at reducing the incidence in the study area.

## 1. Introduction

Bloodstream infections are the leading cause of morbidity and mortality in people of all ages [1], particularly in immunocompromised patients [2]. These infections are frequent and present life-threatening conditions in hospital settings [3, 4]. Globally, bloodstream infection affects about 30 million people leading to 6 million deaths [5], with 3 million newborns and 1.2 million children suffering from sepsis annually [6]. In Eastern African countries, the proportion of patients with bloodstream infection is reported to range from 11% to 28% [1, 7, 8]. In Ghana, bloodstream infection rates as published in hospital and laboratory surveillance reports are estimated at 9.3% to 11.2% [9, 10]. Bloodstream infections are characterized by the presence of viable bacterial or fungal microorganisms in the bloodstream that elicit inflammatory response and often accompanied by alteration of clinical, laboratory, and haemodynamic parameters [11]. These microorganisms may include Gram-negative bacteria such as *Escherichia coli*, *Pseudomonas aeruginosa*, *Klebsiella species*, *Neisseria meningitidis*, and *Haemophilus influenzae*, and Gram-positive bacteria such as coagulase-negative staphylococci (CoNS), *Staphylococcus aureus*, *Streptococcus pneumoniae*, *Streptococcus pyogenes*, *Streptococcus agalactiae*, and *Enterococcus faecium* [12]. The incidence of bloodstream infection is attributed to ageing of patients on admission, increasing number of patients with compromised immunity, and the acquisition of virulence factors by bloodstream pathogens [13, 14] as well as factors linked to infection prevention and control measures and implementation [15]. Bloodstream infections can be categorized into three groups based on its mode of occurrence: in immunocompetent host with intact defenses, in patients at the extremes of life, and in patients affected by pathological conditions putting them at risk to the infections [11]. The symptoms associated with bloodstream infections include, but are not limited to, fever, chills, reduced vascular tone, low blood pressure, change in mental status, hyperventilation, hypothermia, excessive sweating, and likelihood of organ dysfunction [2]. In the determination of causative agents of bloodstream infection, blood cultures are the method of choice because they are highly sensitive and easier to perform [16, 17]. However, this method is not ideal for uncultivable organisms or when antimicrobial treatment has commenced before blood sampling. Although there has been an improvement in public health and medical care in recent times, bacteraemia remains a major cause of sickness and death [18]. There is also a lack of long-term monitoring of bloodstream infection in children and adult in sub-Saharan Africa [1]. Besides, the epidemiological pattern of the causative agents is not static but constantly changing over time [19], necessitating the need for frequent surveillance among the populace. Due to these reasons, this study aimed at describing the epidemiology and antimicrobial susceptibility patterns of bloodstream bacterial infections over a nine-year period at St. Dominic Hospital, Akwatia, in the Eastern Region of Ghana.

## 2. Methods

### 2.1. Study Design and Study Site

This was a retrospective study of 4,489 patients suspected of bloodstream infection over a nine-year period who patronized St. Dominic Hospital from January 2009 to December 2017. The hospital is located in the Denkyembour District of the Eastern Region of Ghana with Akwatia as the district capital. The hospital has a bed capacity of 450 and serves as a referral center for other health facilities located in the district and beyond.

### 2.2. Study Population

Archival records of 4,489 patients with blood culture results available in the hospital's laboratory for review at the time of this study were included. “Commensal bacterium” was considered a contaminant when it was recovered from only one out of the three blood cultures per patient. On the other hand, it was considered pathogenic if it was recovered from multiple venipunctures. Demographic data obtained included age, gender, and department of test requisition.

### 2.3. Study Area

Akwatia is located in the Denkyembour District of the Eastern Region of Ghana. The district was carved out from the Kwabibirem District. The district has a population of 78,841, representing 3.0 percent of the region's total population based on 2010 population and housing census. The females form 50.8% of the total population of the district. The district has a double maxima rainfall regime. The highest monthly rainfall is 414.0 mm.

### 2.4. Statistical Analysis

Data extracted were entered in the Microsoft Excel 2016 spreadsheet for cleaning and validation. Crude incidence and age-standardized incidence were calculated using data from the 2010 Population and Housing Census Denkyembour District Analytical Report as the base population. Population weights were calculated as the total number of people who fell within a specific age group (*n*) divided by the total population in the district (*N*). Year-on-year bacteraemia trends were tested using Cochran–Armitage test for trends. Chi-square test was used to compare the difference in bacteraemia between inpatients and outpatients. A *P* value <0.05 was considered statistically significant. IBM Statistical Package for the Social Sciences version 22.00 (SPSS Inc, Chicago, USA (http://www.spss.com)) was used for data analysis.

### 2.5. Ethical Issues

Approval for this study was obtained from the authority of St. Dominic Hospital. The data retrieved were anonymous and not linked to any patient. Patients' names were not retrieved from the archives.

## 3. Results

The crude incidence of bacteraemia among the population was 77.7 and 72.2 per 10,000 persons for male and female residents, respectively. The age-standardized bacteraemia rates recorded were 77.3 per 10,000 persons for the male population and 75.1 per 10,000 persons for the female population. After age standardization, the overall case density was highest among children under 5 years irrespective of gender. Decreasing incidence of bacteraemia was recorded with increasing age group among both males and females (Table 1).

Within the years under review, 51.4 cases per every 100,000 were diagnosed of bacteraemia. The incidence over the 9-year period was 574.4 and 2.8 per 100,000 per hospital attendance for inpatients and outpatients, respectively. Although not statistically significant, the rate of bacteraemia was observed to have increased from the first 3 years of the review to the last, and this trend was also seen in both inpatients and outpatients (Table 2).

**Figure 1:**
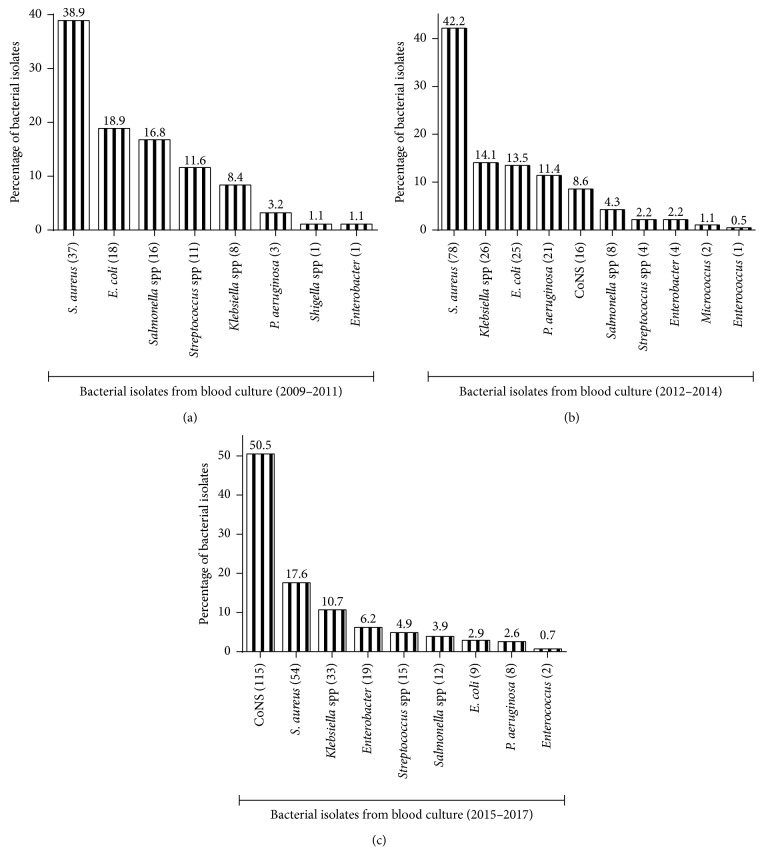
Pattern of bacterial isolates from blood culture at St. Dominic Hospital in Akwatia: (a) 2009–2011, (b) 2012–2014, and (c) 2015–2017. Absolute numbers of isolates are in parentheses.

In general, a changing pattern of bloodstream bacterial infection was observed. *Staphylococcus aureus* representing 38.9% (37/95) in 2009–2011 and 42.2% (78/185) in 2012–2014 was the leading causative agent of bacteraemia in the first two scalar years of the review (Figures 1(a) and 1(b)). Coagulase-negative staphylococcus (CoNS) 50.5% (155/307) in 2015–2017 was the most predominant causative agent of bacteraemia in the last scalar year, followed by *Staphylococcus aureus* (54/37 (17.6%)) (Figure 1(c)). *Klebsiella* spp. and *Enterobacter* infections became increasingly dominant, making the top four infection list, while *Escherichia coli* infection became less dominant (Figure 1).

The modal points of the bacteraemia cases within the 9-year period of the review were observed in the months of October (10.1 per 10,000 persons) and May (8.9 per 10,000 persons). The case difference between the trough month (January = 3 cases per 10,000 persons) and the peak month (October) was in excess of 7 cases per 10,000 persons. The general epidemiological pattern using the crude incidence revealed a rise from the trough month of January, the dry season, through to the peak in October, the rainy season, and then a decline in the incidence onward until December, the dry season (Figure 1(a)). The males recorded a higher crude incidence in most months (February, March, April, May, August, October, and December) compared to the female population (January, June, July, September, and November) (Figure 1(b)).

**Figure 2:**
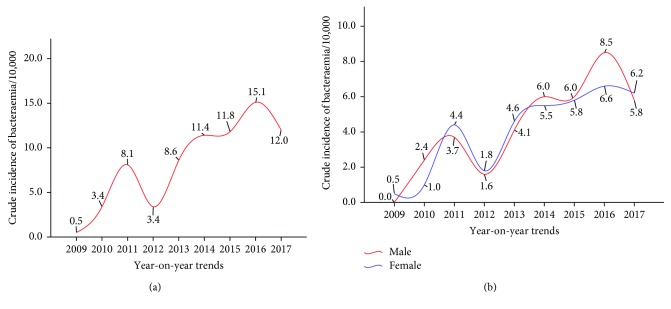
Year-on-year trend of bloodstream bacteraemia reported cases among patients attending St. Dominic Hospital in Akwatia. (a) Trend among all patients/10,000 persons. (b) Gender-specific trend among patients/10,000 persons.

The year-on-year trend of the 9-years period under review revealed an increase in the overall bloodstream bacteraemia from 2013, peaking in 2016 (15.1 per 10,000 persons) (Figure 2(a)). There was gender disparity in the bacteraemia though not prominent through the years; the rate of bacteraemia among the male group overtook the female from 2014 onwards (Figure 2(b)).

Among the bacterial isolates that were identified, 381/1669 (22.8%), 343/689 (49.8%), 207/357 (58%), 294/349 (84.2%), 49/499 (9.8%), 514/747 (68.8%), 36/194 (18.6%), and 140/531 (26.4%) were sensitive to penicillins, cephalosporins, macrolides, quinolones and fluoroquinolones, sulphonamides, aminoglycosides, phenicols, and tetracyclines, respectively. The resistance pattern of these isolates ranged from 0% for nitrofurantoin to 90.2% for the sulphonamides. CoNS, the predominant isolate (171/587 (29.1%)) over the 9-year period, showed susceptibility patterns of 70/75 (93.3%), 92/136 (67.7%), and 108/167 (64.7%) to quinolones and fluoroquinolones, cephalosporins, and aminoglycosides, respectively. The second predominant isolate was *Staphylococcus aureus* (169/587 (28.8%)), which recorded a susceptibility pattern of 52/67 (77.6%), 114/167 (68.3%), and 54/84 (64.3%) to quinolones and fluoroquinolones, aminoglycosides, and cephalosporins, respectively. It was observed that Gram-negative isolates showed higher resistance to penicillins (75%–100%) compared to the Gram-positive isolates (0%–81%). However, the inverse was observed with the aminoglycosides where the Gram-positive isolates rather showed higher resistance (0%–70.6%) compared to the Gram-negative isolates (0%–35.2%) (Tables 3 and 4).

## 4. Discussion

In this study, we observed 587 positive cultures out of the 4,489 suspected cases referred to the hospital's laboratory for culture and sensitivity testing during the 9-year period under review (2009–2017), constituting an overall incidence of 51.4 positive cultures per 100,000 hospital attendance. Among inpatients and outpatients, the incidence of bacteraemia was 574.4 and 2.8 per 100,000 hospital attendance, respectively (Table 2). It is worth mentioning that it was not plausible to draw a direct comparison of our findings to similar studies undertaken previously in Ghana and other West African countries owing to the fact those studies expressed their results as proportions of positive cultures instead of incidence. Notwithstanding, Obeng-Nkrumah et al. [9], Opintan and Newman [10], and Labi et al. [20] recorded percentage bloodstream infections of 9.3%, 11.2%, and 21.9%, respectively, among subpopulations of Ghanaians. In other West African countries, similar percentages of bloodstream infection have been reported in Nigeria [21], Burkina Faso [22], and Côte d'Ivoire [23]. However, Fleischmann et al. [5] in a study to determine the global burden and mortality of sepsis reported incident rates of 288 for hospital-treated sepsis cases and 148 for hospital-treated severe sepsis cases per 100,000 person-years. Although our data are inadequate to validate this assertion, bloodstream infection is reported to be associated with increasing number of immunocompromised patients and increased use of invasive devices [24, 25]. Moreover, the higher incidence of bacterial infection observed among inpatients compared to outpatients in this study also corroborates previous reports [10, 26]. The use of invasive procedures like catheterization, central lines, and mechanical ventilation are some factors proposed to account for higher bloodstream infection rates in hospitalized patients [27].

We found male gender to be more susceptible to bloodstream infections in the present study. Thus, more males (77.7 cases per 10, 000 persons) recorded bacteraemia compared to their female counterparts (72.2 cases per 10,000 persons) (Table 1). Our results add to a growing body of knowledge where male preponderance to bloodstream infections has been reported in the previous studies [7, 23, 28–30]. Some reasons proposed to explain the male gender vulnerability include less frequent hand hygiene practice which could potentially provide enabling environment for large reservoirs of common pathogens responsible for causing bloodstream infections [31, 32], biological makeup of women where oestrogen suppresses the expression of virulence factors of some microorganisms especially *Pseudomonas aeruginosa* [31], and the onset of urinary tract infection in men which often goes undetected, providing fertile environment for the organisms to make their way into the bloodstream [33].

The infectious agents responsible for bloodstream infections vary from country to country with unique geographical peculiarities [3, 23, 34]. The research works of Bouza et al. [25] in Spain, Koupetori et al. [35] in Greece, and Musicha et al. [1] in Malawi attributed bloodstream infections to the predominance of Gram-negative bacteria. However, those results were challenged in the works of Kolonitsiou et al. [36] in Greece, Bassetti et al. [37] in Italy, and Wasihun et al. [8] in Ethiopia, as well as Chiduo et al. [7] in Tanzania who found Gram-positive bacteria to be predominantly responsible for bloodstream infections. In our study, the first two scalar years of the review (2009–2011, 37/95 (38.9%); 2012–2014, 78/185 (42.2%)) revealed *Staphylococcus aureus* as the leading causative agent of bacteraemia (Figures 1(a) and 1(b)) while coagulase-negative staphylococcus (CoNS) (155/307 (50.5%)) was observed to be predominantly responsible for bacteraemia in the last scalar year (2015–2017), followed by *Staphylococcus aureus* (54/307 (17.6%)) (Figure 1(c)). In contrast, however, our results contradict the findings of Labi et al. [20], Obeng-Nkrumah et al. [9], and Opota et al. [17] who reported *Escherichia coli* as the leading cause for bloodstream infections. Staphylococcal predominance may be due to the rise in methicillin-resistant staphylococcus and catheter-related infections, whereas *Escherichia coli*-associated bloodstream infection is usually secondary to hepatobiliary sepsis, abdominal, urinary tract, and surgical tract infections [19].

After age standardization, bacteraemia was highest among children under 5 years irrespective of gender (male vs. female: 50.9 vs. 45.2 per 10,000 persons) compared to the older age groups. The highest age category (<5 years) had the highest incidence (male vs. female: 372.5 vs. 330.9 per 10,000 persons). Thus, an inverse relationship between age and the incidence of bloodstream infection was established in both male and female subpopulations (Table 1). The finding suggests that children were at a greater risk of acquiring bloodstream infections compared to the older age groups. Postulated factors attributed to higher bloodstream infection rate in young patients particularly neonates include immature immune system, poor skin integrity, frequent exposure to healthcare environments, and low socioeconomic status of parents, as well as poor hygiene practices, bottle feeding, and high incidence of delivery at home [12, 38–40]. According to Newman [41], hospital environments abound with lots of nosocomial organisms and this situation could render neonates more susceptible to bloodstream infections.

**Figure 3:**
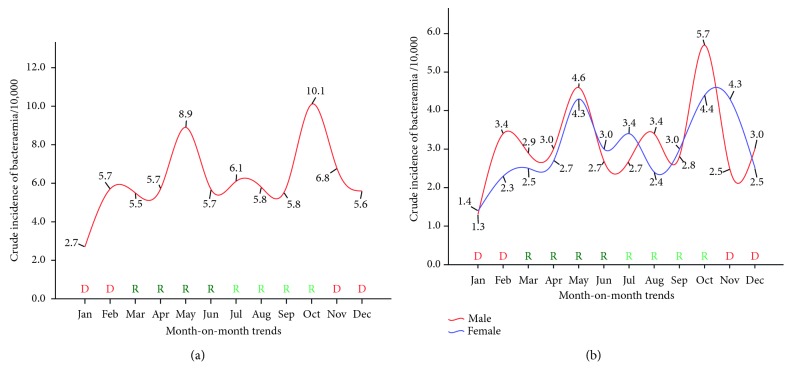
Seasonal variation in bloodstream bacteraemia among patients attending St. Dominic Hospital in Akwatia. D, dry season; R (deep green), major rainy season; R (light green), minor rainy season. (a) Trend among all patients/10,000 persons. (b) Gender-specific trend among patients/10,000 persons.

Seasonal variation is an important determinant of bloodstream infection burden [42, 43]. In Ghana, the months of December-January and May and October coincide with the dry and wet seasons, respectively [44]. In the current study, the epidemiological pattern of bloodstream infection based on the crude incidence revealed an upward trend during the rainy season and a dip in the dry season, with the highest bacteraemia cases recorded in the months of May (8.9 per 10,000 persons) and October (10.1 per 10,000 persons) (Figure 3(a)). Though it was previously reported that higher rates of bloodstream infection directly correlate with increasing temperature [43, 45], our finding seems to contradict those reports. A plausible reason may be due to climatic changes that appear to underlie individuals' susceptibility to bacterial pathogens.

Microbial agents that are associated with bloodstream infections may be modified via antibiotic administration and other factors specific to the patients such as surgical procedures, trauma, or underlying conditions, or by the quality of specimen collection, transport, and culture [46]. Reports of antimicrobial susceptibility patterns of bacterial isolates in body fluids are well documented in the literature worldwide and in the African settings but with varying outcomes [46, 47]. In the present study, the bacterial isolates demonstrated varying resistance patterns to antibiotics ranging from low resistance to quinolones and fluoroquinolones (54/349 (15.5%)) and aminoglycosides (232/747 (31.1%)); moderate resistance to macrolides (149/357 (41.7%)) and cephalosporins (346/689 (50.2%)); and high resistance to tetracyclines (390/531 (73.4%)), penicillins (1282/1669 (76.8%)), and sulphonamides (450/499 (90.2%)). CoNS, the predominant isolate [171/587 (29.1%)] over the 9-year period, showed susceptibility patterns of 70/75 (93.3%), 92/136 (67.7%), and 108/167 (64.7%) to quinolones and fluoroquinolones, cephalosporins, and aminoglycosides, respectively. The second predominant isolate, *Staphylococcus aureus* [169/587 (28.8%)], recorded susceptibility patterns of 52/67 (77.6%), 114/167 (68.3%), and 54/84 (64.3%) to quinolones and fluoroquinolones, aminoglycosides, and cephalosporins, respectively (Tables 3 and 4). Similar results of high resistance to ampicillin (94.4%), cefuroxime (79.0%), and cefotaxime (71.3%) were reported by Agyepong and his colleagues in Ashanti Region [26] and in other parts of the country [48, 49]. The high level of bacterial resistance to antimicrobials in sub-Saharan Africa, including Ghana, is believed to be due to easy availability; thus, they are routinely used in many healthcare facilities and low cost [26]. Moreover, the increasing resistance to the second- and third-generation cephalosporins including cefuroxime and cefotaxime is shown to be associated with the expression or production of extended-spectrum beta-lactamase enzyme [50].

We have identified some limitations worth mentioning in the present study that could potentially influence the interpretations of our findings. Data were retrospectively obtained, and this did not allow for random selection of cases. The study fell short of describing factors that may be associated with bloodstream infections due to unavailability of information, hence limiting our understanding of potential risk factors. Although a considerable number of staphylococcus isolates were included in this study, no data on methicillin resistance test were available. The study was also based on a single-site analysis; hence, findings cannot be generalized. Potentially, changes in hygiene and infection prevention and control practices among the healthcare givers over the period could also affect the findings of this study.

## 5. Conclusion

Bloodstream infection and antimicrobial resistance are high in patients seeking healthcare in Akwatia. This therefore calls for concerted efforts aimed at reducing the burden in the study area.

## Figures and Tables

**Table 1:** Age- and gender-stratified crude incidence of bacteraemia among the population of patients seeking healthcare at St. Dominic Hospital, Akwatia, Ghana.

Parameter	Population		Bacteraemia case		Crude incidence/10,000 persons			Direct age-standardized incidence/10,000 persons	
Age group	Male	Female	Male	Female	Male	Female	P weight	Male	Female
<5 years	5,476	5,228	204	173	372.5	330.9	0.14	50.9	45.2
5–24 years	17,579	17,903	53	54	30.2	30.2	0.45	13.7	13.7
25–44 years	8,927	10,239	19	30	21.3	29.3	0.24	5.2	7.2
45–64 years	5,184	4,719	16	22	30.9	46.6	0.13	3.9	5.9
65–84 years	1,468	1,675	8	8	54.5	47.8	0.04	2.2	1.9
Total	**38,634**	**39,764**	**300**	**287**	**77.7**	**72.2**	**1.00**	**77.3**	**75.1**

Population figures and weight were obtained using the 2010 population figures for the district. P weight, population weight.

**Table 2:** Scalar year-on-year incidence of bacteraemia among patients at St. Dominic Hospital, Akwatia.

Parameter	Total	2009–2011	2012–2014	2015–2017	Cochran–Armitage test for trends
Total hospital attendance	1,141,417	413,426	413,004	314,987	0.377
Positive cultures	587	95	185	307	
Incidence/100,000	51.4	23.0	44.8	97.5	

Population of inpatients	97,150	35,691	33,557	27,902	0.213
Positive cultures	558	89	173	296	
Incidence/100,000	574.4	249.4	515.5	1,060.9	

Population of outpatients	1,044,267	377,735	379,447	287,085	0.931
Positive cultures	29	6	12	11	
Incidence/100,000	2.8	1.6	3.2	3.8	

Chi-square *P* value	**0.001**	0.200	**0.011**	0.189	

*P* values are significant at <0.05.

**Table 3:** Antimicrobial susceptibility pattern of bacterial isolates from blood culture at St. Dominic Hospital in Akwatia.

Bacterial isolate	Pattern	Penicillin	Cephalosporins	Macrolides	Quinolones and fluoroquinolones	Sulphonamides
		*n* (%)	*n* (%)	*n* (%)	*n* (%)	*n* (%)
*S. aureus* ^*∗*^	S	116 (17.3)	54 (64.3)	104 (64.2)	52 (77.6)	24 (14.8)
	I	5 (0.7)	0 (0.0)	1 (0.6)	0 (0.0)	0 (0.0)
	R	549 (81.9)	30 (35.7)	57 (35.2)	15 (22.4)	138 (85.2)

*E. coli*	S	10 (14.1)	35 (32.1)	0 (0.0)	48 (87.3)	4 (10.5)
	I	0 (0.0)	0 (0.0)	0 (0.0)	0 (0.0)	0 (0.0)
	R	61 (85.9)	74 (67.9)	1 (100.0)	7 (12.7)	34 (89.5)

*P. aeruginosa*	S	1 (25.0)	5 (33.3)	0 (0.0)	48 (96.0)	0 (0.0)
	I	0 (0.0)	0 (0.0)	0 (0.0)	0 (0.0)	0 (0.0)
	R	3 (75.0)	10 (66.7)	0 (0.0)	2 (4.0)	0 (0.0)

*Streptococcus* spp.^*∗*^	S	30 (29.7)	21 (72.4)	14 (51.9)	9 (69.2)	1 (3.8)
	I	0 (0.0)	0 (0.0)	0 (0.0)	0 (0.0)	0 (0.0)
	R	71 (70.3)	8 (27.6)	13 (48.1)	4 (30.8)	25 (96.2)

*Klebsiella* spp.	S	8 (9.0)	40 (25.0)	0 (0.0)	31 (62.0)	3 (5.7)
	I	0 (0.0)	0 (0.0)	0 (0.0)	0 (0.0)	0 (0.0)
	R	81 (91.0)	120 (75.0)	0 (0.0)	619 (38.0)	50 (94.3)

*Shigella* spp.	S	0 (0.0)	0 (0.0)	0 (0.0)	1 (100.0)	0 (0.0)
	I	0 (0.0)	0 (0.0)	0 (0.0)	0 (0.0)	0 (0.0)
	R	1 (100.0)	2 (100.0)	0 (0.0)	0 (0.0)	1 (100.0)

*Enterobacter*	S	6 (15.8)	16 (25.0)	0 (0.0)	16 (88.9)	2 (9.1)
	I	0 (0.0)	0 (0.0)	0 (0.0)	1 (5.6)	0 (0.0)
	R	32 (84.2)	48 (75.0)	0 (0.0)	1 (5.6)	20 (90.9)

*Enterococcus* ^*∗*^	S	2 (15.4)	0 (0.0)	1 (50.0)	0 (0.0)	0 (0.0)
	I	0 (0.0)	0 (0.0)	0 (0.0)	0 (0.0)	0 (0.0)
	R	11 (84.6)	2 (100.0)	1 (50.0)	1 (100.0)	3 (100.0)

*Micrococcus* ^*∗*^	S	0 (0.0)	0 (0.0)	0 (0.0)	0 (0.0)	0 (0.0)
	I	0 (0.0)	0 (0.0)	0 (0.0)	0 (0.0)	0 (0.0)
	R	0 (0.0)	0 (0.0)	0 (0.0)	0 (0.0)	0 (0.0)

CoNS^*∗*^	S	198 (31.2)	92 (67.6)	88 (53.3)	70 (93.3)	11 (6.9)
	I	1 (0.2)	0 (0.0)	0 (0.0)	0 (0.0)	0 (0.0)
	R	436 (68.7)	44 (32.4)	77 (46.7)	5 (6.7)	148 (93.1)

*Salmonella* spp.	S	10 (21.3)	80 (90.9)	0 (0.0)	19 (100.0)	4 (11.4)
	I	0 (0.0)	0 (0.0)	0 (0.0)	0 (0.0)	0 (0.0)
	R	37 (78.7)	8 (9.1)	0 (0.0)	0 (0.0)	31 (88.6)

Total	S	381 (22.8)	343 (49.8)	207 (58.0)	294 (84.2)	49 (9.8)
	I	6 (0.4)	0 (0.0)	1 (0.3)	1 (0.3)	0 (0.0)
	R	1282 (76.8)	346 (50.2)	149 (41.7)	54 (15.5)	450 (90.2)

Data are presented as numbers and percentages in parentheses. ^*∗*^Gram-positive isolates. CoNS, coagulase-negative staphylococcus; S, susceptible; I, intermediately resistant; R, resistant.

**Table 4:** Antimicrobial susceptibility pattern of bacterial isolates from blood culture at St. Dominic Hospital in Akwatia.

Bacterial isolate	Pattern	Aminoglycosides	Phenicols	Nitrofurantoin	Glycopeptides	Tetracyclines
		*n* (%)	*n* (%)	*n* (%)	*n* (%)	*n* (%)
*S. aureus* ^*∗*^	S	114 (68.3)	1 (16.7)	0 (0.0)	2 (100.0)	61 (36.7)
	I	0 (0.0)	0 (0.0)	0 (0.0)	0 (0.0)	1 (0.6)
	R	53 (31.7)	5 (83.3)	0 (0.0)	0 (0.0)	104 (62.7)

*E. coli*	S	69 (75.0)	16 (29.1)	0 (0.0)	0 (0.0)	8 (16.7)
	I	0 (0.0)	0 (0.0)	0 (0.0)	0 (0.0)	0 (0.0)
	R	23 (25.0)	39 (70.9)	0 (0.0)	0 (0.0)	40 (83.3)

*P. aeruginosa*	S	51 (82.3)	0 (0.0)	0 (0.0)	0 (0.0)	1 (33.3)
	I	0 (0.0)	0 (0.0)	0 (0.0)	0 (0.0)	0 (0.0)
	R	11 (17.7)	2 (100.0)	0 (0.0)	0 (0.0)	2 (66.7)

*Streptococcus* spp.^*∗*^	S	10 (29.4)	0 (0.0)	0 (0.0)	0 (0.0)	5 (18.5)
	I	0 (0.0)	0 (0.0)	0 (0.0)	0 (0.0)	0 (0.0)
	R	24 (70.6)	1 (100.0)	0 (0.0)	0 (0.0)	22 (81.5)

*Klebsiella* spp.	S	80 (64.0)	9 (13.0)	1 (100.0)	0 (0.0)	5 (8.1)
	I	1 (0.8)	0 (0.0)	0 (0.0)	0 (0.0)	0 (0.0)
	R	44 (35.2)	60 (87.0)	0 (0.0)	0 (0.0)	57 (91.9)

*Shigella* spp.	S	2 (100.0)	1 (100.0)	0 (0.0)	0 (0.0)	0 (0.0)
	I	0 (0.0)	0 (0.0)	0 (0.0)	0 (0.0)	0 (0.0)
	R	0 (0.0)	0 (0.0)	0 (0.0)	0 (0.0)	1 (100.0)

*Enterobacter*	S	25 (64.1)	3 (13.6)	0 (0.0)	0 (0.0)	4 (16.7)
	I	0 (0.0)	0 (0.0)	0 (0.0)	0 (0.0)	0 (0.0)
	R	14 (35.9)	19 (86.4)	0 (0.0)	0 (0.0)	20 (83.3)

*Enterococcus* ^*∗*^	S	1 (33.3)	0 (0.0)	0 (0.0)	0 (0.0)	2 (66.7)
	I	0 (0.0)	0 (0.0)	0 (0.0)	0 (0.0)	0 (0.0)
	R	2 (66.7)	0 (0.0)	0 (0.0)	0 (0.0)	1 (33.3)

*Micrococcus* ^*∗*^	S	0 (0.0)	0 (0.0)	0 (0.0)	0 (0.0)	0 (0.0)
	I	0 (0.0)	0 (0.0)	0 (0.0)	0 (0.0)	0 (0.0)
	R	0 (0.0)	0 (0.0)	0 (0.0)	0 (0.0)	0 (0.0)

CoNS^*∗*^	S	108 (64.7)	1 (25.0)	0 (0.0)	0 (0.0)	42 (25.8)
	I	0 (0.0)	0 (0.0)	0 (0.0)	0 (0.0)	0 (0.0)
	R	59 (35.3)	3 (75.0)	0 (0.0)	1 (100.0)	121 (74.2)

*Salmonella* spp.	S	54 (96.4)	5 (14.7)	0 (0.0)	0 (0.0)	12 (35.3)
	I	0 (0.0)	0 (0.0)	0 (0.0)	0 (0.0)	0 (0.0)
	R	2 (3.6)	29 (85.3)	0 (0.0)	0 (0.0)	22 (64.7)

Total	S	514 (68.8)	36 (18.6)	1 (100.0)	2 (66.7)	140 (26.4)
	I	1 (0.1)	0 (0.0)	0 (0.0)	0 (0.0)	1 (0.2)
	R	232 (31.1)	158 (81.4)	0 (0.0)	1 (33.3)	390 (73.4)

Data are presented as numbers and percentages in parentheses. ^*∗*^Gram-positive isolates. CoNS, coagulase-negative staphylococcus; S, susceptible; I, intermediately resistant; R, resistant.

## Data Availability

The data used to support the findings of this study are available from the corresponding author upon request.

## Supplementary Materials

Supplementary Materials Table 4A: antimicrobial susceptibility pattern of bacterial isolates from blood culture at St. Dominic Hospital in Akwatia.
