# Supplementary material for: The Epidemiology of Bloodstream Infections and Antimicrobial Susceptibility Patterns: A Nine-Year Retrospective Study at St. Dominic Hospital, Akwatia, Ghana
Source: J Trop Med. 2019 Sep 19;2019:6750864. doi: 10.1155/2019/6750864 (PMC6770298; doi:10.1155/2019/6750864)
Supplement: Supplementary Materials — Table 4A: antimicrobial susceptibility pattern of bacterial isolates from blood culture at St. Dominic Hospital in Akwatia. [file 6750864.f1.pdf]

Table 4A: **Antimicrobial susceptibility pattern of bacteria isolates from blood culture at the St. Dominic Hospital in Akwatia**

		PENICILLINS								
**Bacteria isolate**	**Pattern**	**COX n(%)**	**AMX n(%)**	**PEN n(%)**	**AMP n(%)**	**TZP n(%)**	**AUG n(%)**	**FLX n(%)**	**CXC n(%)**	**AS n(%)**
S. aureus	S	3(100.0)	0(0.0)	1(0.6)	6(3.7)	0(0.0)	78(81.3)	11(17.7)	17(9.1)	0(0.0)
	I	0(0.0)	0(0.0)	1(0.6)	0(0.0)	0(0.0)	0(0.0)	2(3.2)	2(1.1)	0(0.0)
	R	0(0.0)	0(0.0)	158(98.8)	157(96.3)	0(0.0)	18(18.8)	49(79.0)	167(89.8)	0(0.0)
E. coli	S	0(0.0)	0(0.0)	0(0.0)	1(2.6)	4(33.3)	2(40.0)	0(0.0)	0(0.0)	3(25.0)
	I	0(0.0)	0(0.0)	0(0.0)	0(0.0)	0(0.0)	0(0.0)	0(0.0)	0(0.0)	0(0.0)
	R	1(100.0)	0(0.0)	1(100.0)	38(97.4)	8(66.7)	3(60.0)	1(100.0)	0(0.0)	9(75.0)
P. aeruginosa	S	0(0.0)	0(0.0)	0(0.0)	0(0.0)	0(0.0)	0(0.0)	0(0.0)	0(0.0)	1(33.3)
	I	0(0.0)	0(0.0)	0(0.0)	0(0.0)	0(0.0)	0(0.0)	0(0.0)	0(0.0)	0(0.0)
	R	0(0.0)	0(0.0)	0(0.0)	0(0.0)	1(100.0)	0(0.0)	0(0.0)	0(0.0)	2(66.7)
Streptococcus spp	S	1(100.0)	0(0.0)	2(7.7)	4(15.4)	0(0.0)	14(77.8)	2(15.4)	7(41.2)	0(0.0)
	I	0(0.0)	0(0.0)	0(0.0)	0(0.0)	0(0.0)	0(0.0)	0(0.0)	0(0.0)	0(0.0)
	R	0(0.0)	0(0.0)	24(92.3)	22(84.6)	0(0.0)	4(22.2)	11(84.6)	10(58.8)	0(0.0)
Klebsiella spp	S	0(0.0)	0(0.0)	0(0.0)	1(1.8)	2(28.6)	5(33.3)	0(0.0)	0(0.0)	0(0.0)
	I	0(0.0)	0(0.0)	0(0.0)	0(0.0)	0(0.0)	0(0.0)	0(0.0)	0(0.0)	0(0.0)
	R	0(0.0)	0(0.0)	1(100.0)	56(98.2)	5(71.4)	10(66.7)	0(0.0)	2(100.0)	7(100.0)
Shigella spp	S	0(0.0)	0(0.0)	0(0.0)	0(0.0)	0(0.0)	0(0.0)	0(0.0)	0(0.0)	0(0.0)
	I	0(0.0)	0(0.0)	0(0.0)	0(0.0)	0(0.0)	0(0.0)	0(0.0)	0(0.0)	0(0.0)
	R	0(0.0)	0(0.0)	0(0.0)	1(100.0)	0(0.0)	0(0.0)	0(0.0)	0(0.0)	0(0.0)
Enterobacter	S	0(0.0)	0(0.0)	0(0.0)	0(0.0)	0(0.0)	6(54.5)	0(0.0)	0(0.0)	0(0.0)
	I	0(0.0)	0(0.0)	0(0.0)	0(0.0)	0(0.0)	0(0.0)	0(0.0)	0(0.0)	0(0.0)
	R	0(0.0)	0(0.0)	0(0.0)	23(100.0)	0(0.0)	5(45.5)	0(0.0)	4(100.0)	0(0.0)
Enterococcus	S	0(0.0)	0(0.0)	0(0.0)	0(0.0)	0(0.0)	2(66.7)	0(0.0)	0(0.0)	0(0.0)
	I	0(0.0)	0(0.0)	0(0.0)	0(0.0)	0(0.0)	0(0.0)	0(0.0)	0(0.0)	0(0.0)
	R	0(0.0)	0(0.0)	3(100.0)	3(100.0)	0(0.0)	1(33.3)	2(100.0)	2(100.0)	0(0.0)
Micrococcus	S	0(0.0)	0(0.0)	0(0.0)	0(0.0)	0(0.0)	0(0.0)	0(0.0)	0(0.0)	0(0.0)
	I	0(0.0)	0(0.0)	0(0.0)	0(0.0)	0(0.0)	0(0.0)	0(0.0)	0(0.0)	0(0.0)
	R	0(0.0)	0(0.0)	0(0.0)	0(0.0)	0(0.0)	0(0.0)	0(0.0)	0(0.0)	0(0.0)
CoNS	S	6(100.0)	0(0.0)	3(2.1)	16(10.6)	0(0.0)	139(89.7)	30(33.7)	4(4.5)	0(0.0)
	I	0(0.0)	0(0.0)	0(0.0)	0(0.0)	0(0.0)	1(0.6)	0(0.0)	0(0.0)	0(0.0)
	R	0(0.0)	6(100.0)	137(97.9)	135(89.4)	0(0.0)	15(9.7)	59(66.3)	84(95.5)	0(0.0)
Salmonella spp	S	0(0.0)	0(0.0)	0(0.0)	1(3.1)	0(0.0)	9(69.2)	0(0.0)	0(0.0)	0(0.0)
	I	0(0.0)	0(0.0)	0(0.0)	0(0.0)	0(0.0)	0(0.0)	0(0.0)	0(0.0)	0(0.0)
	R	0(0.0)	0(0.0)	0(0.0)	31(96.9)	0(0.0)	4(30.8)	0(0.0)	2(100.0)	0(0.0)

Data is presented as figure and percentages in parentheses

Bacteria isolate	Pattern	Cephalosporins							Macrolides	
		CTX n(%)	CRO n(%)	CTR n(%)	CRX n(%)	CAZ n(%)	CF n(%)	CTL n(%)	ERY n(%)	AZM n(%)
S.aureus	S	2(40.0)	0(0.0)	1(25.0)	51(68.0)	0(0.0)	0(0.0)	0(0.0)	104(64.2)	0(0.0)
	I	0(0.0)	0(0.0)	0(0.0)	0(0.0)	0(0.0)	0(0.0)	0(0.0)	1(0.6)	0(0.0)
	R	3(60.0)	0(0.0)	3(75.0)	24(32.0)	0(0.0)	0(0.0)	0(0.0)	57(35.2)	0(0.0)
E. coli	S	13(35.1)	0(0.0)	9(37.5)	9(24.3)	0(0.0)	3(30.0)	1(100.0)	0(0.0)	0(0.0)
	I	0(0.0)	0(0.0)	0(0.0)	0(0.0)	0(0.0)	0(0.0)	0(0.0)	0(0.0)	0(0.0)
	R	24(64.9)	0(0.0)	15(62.5)	28(75.7)	0(0.0)	7(70.0)	0(0.0)	1(100.0)	0(0.0)
P. aeruginosa	S	2(66.7)	0(0.0)	0(0.0)	0(0.0)	3(27.3)	0(0.0)	0(0.0)	0(0.0)	0(0.0)
	I	0(0.0)	0(0.0)	0(0.0)	0(0.0)	0(0.0)	0(0.0)	0(0.0)	0(0.0)	0(0.0)
	R	1(33.3)	0(0.0)	0(0.0)	0(0.0)	8(72.7)	1(100.0)	0(0.0)	0(0.0)	0(0.0)
Streptococcus spp	S	1(100.0)	0(0.0)	4(100.0)	15(71.4)	1(33.3)	0(0.0)	0(0.0)	14(51.9)	0(0.0)
	I	0(0.0)	0(0.0)	0(0.0)	0(0.0)	0(0.0)	0(0.0)	0(0.0)	0(0.0)	0(0.0)
	R	0(0.0)	0(0.0)	0(0.0)	6(28.6)	2(66.7)	0(0.0)	0(0.0)	13(48.1)	0(0.0)
Klebsiella spp	S	14(26.4)	1(100.0)	14(31.1)	11(20.8)	0(0.0)	0(0.0)	0(0.0)	0(0.0)	0(0.0)
	I	0(0.0)	0(0.0)	0(0.0)	0(0.0)	0(0.0)	0(0.0)	0(0.0)	0(0.0)	0(0.0)
	R	39(73.6)	0(0.0)	31(68.9)	42(79.2)	1(100.0)	6(100.0)	1(100.0)	0(0.0)	0(0.0)
Shigella spp	S	0(0.0)	0(0.0)	0(0.0)	0(0.0)	0(0.0)	0(0.0)	0(0.0)	0(0.0)	0(0.0)
	I	0(0.0)	0(0.0)	0(0.0)	0(0.0)	0(0.0)	0(0.0)	0(0.0)	0(0.0)	0(0.0)
	R	1(100.0)	0(0.0)	0(0.0)	1(100.0)	0(0.0)	0(0.0)	0(0.0)	0(0.0)	0(0.0)
Enterobacter	S	4(17.4)	0(0.0)	7(35.0)	5(23.8)	0(0.0)	0(0.0)	0(0.0)	0(0.0)	0(0.0)
	I	0(0.0)	0(0.0)	0(0.0)	0(0.0)	0(0.0)	0(0.0)	0(0.0)	0(0.0)	0(0.0)
	R	19(82.6)	0(0.0)	13(65.0)	16(76.2)	0(0.0)	0(0.0)	0(0.0)	0(0.0)	0(0.0)
Enterococcus	S	0(0.0)	0(0.0)	0(0.0)	0(0.0)	0(0.0)	0(0.0)	0(0.0)	1(50.0)	0(0.0)
	I	0(0.0)	0(0.0)	0(0.0)	0(0.0)	0(0.0)	0(0.0)	0(0.0)	0(0.0)	0(0.0)
	R	0(0.0)	0(0.0)	0(0.0)	2(100.0)	0(0.0)	0(0.0)	0(0.0)	1(50.0)	0(0.0)
Micrococcus	S	0(0.0)	0(0.0)	0(0.0)	0(0.0)	0(0.0)	0(0.0)	0(0.0)	0(0.0)	0(0.0)
	I	0(0.0)	0(0.0)	0(0.0)	0(0.0)	0(0.0)	0(0.0)	0(0.0)	0(0.0)	0(0.0)
	R	0(0.0)	0(0.0)	0(0.0)	0(0.0)	0(0.0)	0(0.0)	0(0.0)	0(0.0)	0(0.0)
CoNS	S	5(27.8)	0(0.0)	5(71.4)	82(73.9)	0(0.0)	0(0.0)	0(0.0)	83(52.2)	5(83.3)
	I	0(0.0)	0(0.0)	0(0.0)	0(0.0)	0(0.0)	0(0.0)	0(0.0)	0(0.0)	0(0.0)
	R	13(72.2)	0(0.0)	2(28.6)	29(26.1)	0(0.0)	0(0.0)	0(0.0)	76(47.8)	1(16.7)
Salmonella spp	S	32(94.1)	0(0.0)	21(100.0)	27(81.8)	0(0.0)	0(0.0)	0(0.0)	0(0.0)	0(0.0)
	I	0(0.0)	0(0.0)	0(0.0)	0(0.0)	0(0.0)	0(0.0)	0(0.0)	0(0.0)	0(0.0)
	R	2(5.9)	0(0.0)	0(0.0)	6(18.2)	0(0.0)	0(0.0)	0(0.0)	0(0.0)	0(0.0)
